# Determinants of the cuff-leak test: a physiological study

**DOI:** 10.1186/cc3012

**Published:** 2004-11-29

**Authors:** George Prinianakis, Christina Alexopoulou, Eutichis Mamidakis, Eumorfia Kondili, Dimitris Georgopoulos

**Affiliations:** 1 Intensive Care Medicine Department, University of Crete, University Hospital of Heraklion, Heraklion, Crete, Greece; 2 Director, Intensive Care Medicine Department, University of Crete, University Hospital of Heraklion, Heraklion, Crete, Greece

**Keywords:** compliance, inspiratory flow, mechanical ventilation, post-extubation stridor, resistance

## Abstract

**Introduction:**

The cuff-leak test has been proposed as a simple method to predict the occurrence of post-extubation stridor. The test is performed by cuff deflation and measuring the expired tidal volume a few breaths later (*V*_T_). The leak is calculated as the difference between *V*_T _with and without a deflated cuff. However, because the cuff remains deflated throughout the respiratory cycle a volume of gas may also leak during inspiration and therefore this method (conventional) measures the total leak consisting of an inspiratory and expiratory component. The aims of this physiological study were, first, to examine the effects of various variables on total leak and, second, to compare the total leak with that obtained when the inspiratory component was eliminated, leaving only the expiratory leak.

**Methods:**

In 15 critically ill patients mechanically ventilated on volume control mode, the cuff-leak volume was measured randomly either by the conventional method (Leak_conv_) or by deflating the cuff at the end of inspiration and measuring the *V*_T _of the following expiration (Leak_pause_). To investigate the effects of respiratory system mechanics and inspiratory flow, cuff-leak volume was studied by using a lung model, varying the cross-sectional area around the endotracheal tube and model mechanics.

**Results:**

In patients Leak_conv _was significantly higher than Leak_pause_, averaging 188 ± 159 ml (mean ± SD) and 61 ± 75 ml, respectively. In the model study Leak_conv _increased significantly with decreasing inspiratory flow and model compliance. Leak_pause _and Leak_conv _increased slightly with increasing model resistance, the difference being significant only for Leak_pause_. The difference between Leak_conv _and Leak_pause _increased significantly with decreasing inspiratory flow (*V*'_I_) and model compliance and increasing cross-sectional area around the tube.

**Conclusion:**

We conclude that the cross-sectional area around the endotracheal tube is not the only determinant of the cuff-leak test. System compliance and inspiratory flow significantly affect the test, mainly through an effect on the inspiratory component of the total leak. The expiratory component is slightly influenced by respiratory system resistance.

## Introduction

In mechanically ventilated patients the frequency of post-extubation stridor is estimated to range between 4% and 22% [1-3]. Post-extubation stridor is usually due to laryngeal edema or decreased cross-sectional area of trachea, although vocal-cord dysfunction and overdose of sedative drugs may be also the cause. Nevertheless, this complication may result in emergency re-intubation in rather difficult circumstances with increased morbidity and mortality. The cuff-leak test has been proposed as a simple method of predicting the occurrence of this complication [4-7]. This test consists of deflating the balloon cuff of the endotracheal tube to assess the air leak around the tube during expiration by measuring the expiratory tidal volume with and without a deflated cuff [4-6]. A relatively large difference between these two values indicates that the cross-sectional area of the tracheal and/or upper airways is large enough to render the occurrence of post-extubation stridor, and therefore the possibility of re-intubation due to airway obstruction, unlikely [4-7]. Obviously the cuff-leak test is not useful if vocal cord dysfunction or overdose of sedative drugs is the cause of post-extubation stridor.

Typically the cuff-leak test is performed during volume control ventilation (using a tidal volume of 10 ml/kg) by deflating the cuff, whereas the expired tidal volume is measured a few breaths later [4-7]. The leak is calculated as the difference between the expiratory tidal volume with and without a deflated cuff [4-7]. However, because most ventilators in the intensive care unit do not compensate for leaks, it is possible that during inspiration with a deflated cuff a portion of the total amount of the predetermined volume given by the ventilator may leak around the endotracheal tube. In this case, the difference between expiratory tidal volume with and without a deflated cuff represents a total leak consisting of an inspiratory and an expiratory component. This total leak may depend on various factors such as the cross-sectional area around the endotracheal tube, inspiratory flow and respiratory system mechanics, which may affect either the inspiratory component or the expiratory component or both, therefore contributing to the poor performance of the cuff-leak test in identifying patients with post-extubation stridor, reported by some studies [8]. The aims of this physiological study were, first, to examine the effects of various variables, such as cross-sectional area around the endotracheal tube, inspiratory flow and respiratory system mechanics on total leak, and second, to compare the total leak with that obtained when the inspiratory component was eliminated, leaving only the expiratory leak. The inspiratory leak was eliminated by deflating the cuff at end-inspiration, a manoeuvre that guarantees that the ventilator delivers all the predetermined gas volume into the lung.

## Methods

### Clinical study

Fifteen mechanically ventilated patients (aged 65 ± 19 years [mean ± SD]; seven males, eight females) were prospectively studied. All were orotracheally intubated (low-pressure cuff endotracheal tube, diameter 8.0 ± 0.5 mm, tube length 28 ± 1 mm), hemodynamically stable without vasoactive drugs, lightly sedated with propofol and with a PaO_2_/F_i_O_2 _of more than 250 mmHg. The study was approved by the Hospital Ethics Committee, and informed consent was obtained from the patients or their families.

Flow (*V*') at the airway opening was measured with a heated pneumotachograph (model 3700; Hans-Rudolf, Kansas City, KS, USA) and a differential pressure transducer (Micro-Switch 140PC; Honeywell Ltd, Montreal, Ontario, Canada), both placed between the endotracheal tube and the Y-piece of the ventilator. Flow was electronically integrated to provide volume. Airway pressure (*P*_aw_; Micro-Switch 140PC; Honeywell Ltd) was measured from a side port between the pneumotachograph and the endotracheal tube. Each signal was sampled at 150 Hz (Windaq Instruments Inc., Akron, OH, USA) and stored on a computer disk for later analysis.

Initially the patients were placed on volume control mode (Puritan-Bennett 840, Lenexa, KS, USA) with no flow compensation, heavily sedated (propofol–fentanyl) to achieve a Ramsay scale of 6 and paralyzed with *cis*-atracurium. Inactivity of respiratory muscles was confirmed with the use of standard criteria [9]. Tidal volume (*V*_T_) was set to 10 ml/kg given with a constant inspiratory flow rate of 1 litre/s. No end-inspiratory pause was applied. External positive end-expiratory pressure (PEEP) was set to zero while ventilator frequency was adjusted such as to achieve zero intrinsic PEEP, confirmed by end-expiratory occlusion [10].

When the patients were stable on volume control, the (baseline) expiratory *V*_T _was measured by averaging five consecutive breaths (*V*_T,baseline_). The absence of a leak was verified by an end-inspiratory occlusion of 10 s and observing a constant *P*_aw _after 3 s of occlusion. Thereafter, the cuff-leak test was performed randomly, either using the conventional method or by deflating the cuff at the end of a 3 s end-inspiratory pause. The conventional method consisted of balloon cuff deflation and measuring the expiratory tidal volume four breaths later (*V*_T,defl_). Five such trials were performed to obtain an average value of *V*_T,defl_. The difference between *V*_T,baseline _and *V*_T,defl _was defined as the cuff-leak volume obtained by the conventional method (Leak_conv_). When the cuff was deflated at the end of the end-inspiratory pause only the following expiratory tidal volume was measured (*V*_T,pause_). Again five such trials were performed. The difference between *V*_T,baseline _and *V*_T,pause _was defined as the cuff-leak volume obtained by deflating the cuff during end-inspiratory pause (Leak_pause_).

The mechanics of the respiratory system were measured by using the occlusion technique [10-12]. In each patient at least five breaths with a satisfactory plateau were analyzed and the mean values were reported. Respiratory system static inflation end-inspiratory compliance (*C*_rs_), minimum (*R*_int_) and maximum (*R*_rs_) resistance of the respiratory system and the difference between *R*_rs _and *R*_int _(Δ*R*) were computed according to standard formulas and procedures [11,12].

In all patients ΔLeak was calculated as the difference between Leak_conv _and Leak_pause_. Assuming that the difference between peak inspiratory *P*_aw _(Δ*P*_aw,peak_) between methods was entirely due to different end-inspiratory lung volume, the predicted ΔLeak was calculated by the product of Δ*P*_aw,peak _and *C*_rs_.

### Lung model study

To examine the effects of various variables on cuff-leak volume measurement, a two-chamber test lung (Michigan Instruments Inc., Grand Rapids, MI, USA) was used [13]. Each chamber was connected to a common tube representing the trachea by a tube with varying resistance. The compliance of each chamber was also variable. The two chambers were connected to a ventilator (Puritan-Bennett 840) via a cuffed endotracheal tube 8 mm in diameter inserted into the common tube. Small plastic bands were inserted between the endotracheal tube and the common tube to create controlled leaks when the balloon cuff was deflated. Two levels of leak were created, simulating two different cross-sectional areas around the endotracheal tube (large and small). The cross-sectional area around the endotracheal tube was quantified by cuff deflation during the end-inspiratory pause time and observation of the rate of pressure drop when an inspired tidal volume of l litre was used and total model compliance was 50 ml/cmH_2_O. The rate of pressure decrease was about 10 and 5 cmH_2_O/s with large and small cross-sectional areas, respectively. The absence of leak with the cuff inflated was confirmed by end-inspiratory occlusion and demonstration of a constant plateau *P*_aw_.

*V*_T _was set at 0.6 litre (given with constant flow rate) and external PEEP to zero throughout. Ventilator frequency was adjusted so that no dynamic hyperinflation was observed. The absence of dynamic hyperinflation was verified by end-expiratory occlusion and no intrinsic PEEP demonstration [10]. Two protocols were performed. In the first (protocol A), the effects of inspiratory flow (*V*'_I_) on cuff-leak volume measurement as well as the interaction between *V*'_I_, cross-sectional area around the endotracheal tube and model mechanics were studied. At small and large cross-sectional area around the endotracheal tube and three combinations of model mechanics, representing normal (model airway resistance, *R *= 8 cmH_2_O/litre per second; model airway compliance, *C *= 50 ml/cmH_2_O), restrictive (*R *= 8 cmH_2_O/litre per second, *C *= 20 ml/cmH_2_O) and obstructive pattern (*R *= 16 cmH_2_O/litre per second, *C *= 100 ml/cmH_2_O), *V*'_I _was varied between 0.6 and 1 litre/s and cuff-leak volume was measured either by the conventional method or by deflating the cuff at the end of a 3 s end-inspiratory pause as described above. The effects of model mechanics on cuff-leak volume were further studied in a separate protocol (protocol B). At a constant cross-sectional area around the endotracheal tube (large) and an inspiratory flow of 0.6, each method of cuff-leak volume measurement was studied at three levels of *R *and *C*, resulting in nine combinations of system mechanics (*R *= 8, 16 and 32 cmH_2_O/litre per second and *C *= 20, 50 and 100 ml/cmH_2_O). Similarly to protocol A, at each combination of model mechanics the cuff-leak volume was measured either by the conventional method or by deflating the cuff at the end of a 3 s end-inspiratory pause.

Data were analyzed with a paired *t*-test and a multi-factorial analysis of variance for repeated measurements, where appropriate. When the *F *value was significant, Tukey's test was used to identify significant differences. Linear regression analysis was performed with the least-squares method. *P *< 0.05 was considered statistically significant. Data are expressed as means ± SD. In the lung model study, means ± SD for the variables were determined from a total of 10 measurements.

## Results

### Clinical study

**Figure 1:**
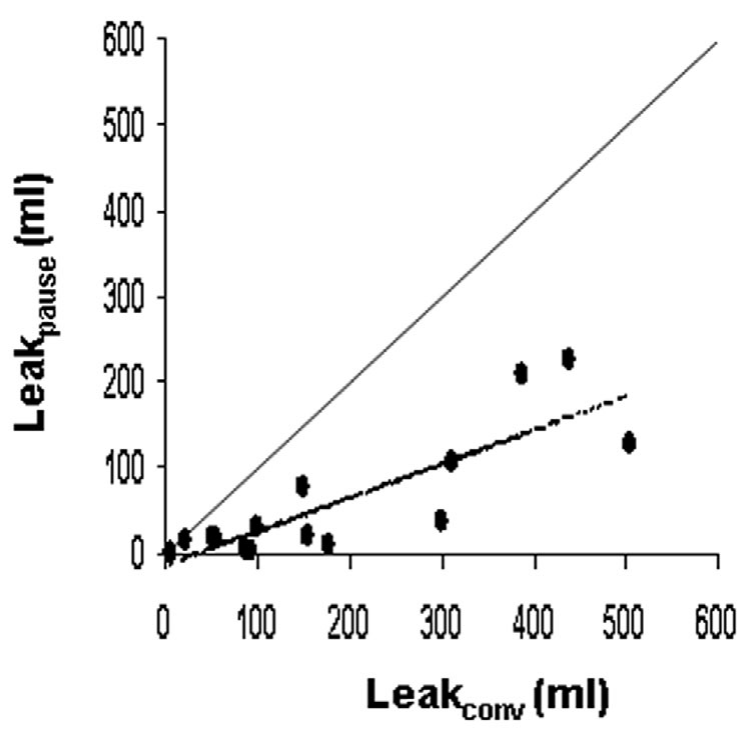
Clinical study. Individual cuff-leak volume was measured when the cuff remained deflated both during inspiration and expiration (conventional method, Leak_conv_) and when the cuff was deflated at the end of 3 s of inspiratory pause (Leak_pause_). Notice that in all patients Leak_conv _is higher than Leak_pause_. Solid line, line of identity; broken line, regression line.

Baseline ventilator settings and respiratory mechanics are shown in Table 1. When the cuff remained deflated throughout the respiratory cycle, *P*_aw,peak _of the analyzed breaths (24.0 ± 6.6 cmH_2_O) was significantly lower than that of the breath in which the cuff was deflated at the end of the inspiratory pause (26.7 ± 7.1 cmH_2_O); the mean Δ*P*_aw,peak _averaged 2.6 ± 2.6 cmH_2_O (range 0.5–8.2 cmH_2_O). As expected, *P*_aw,peak _of the breaths in which the cuff was deflated at the end of the inspiratory pause was similar to the corresponding value of the baseline. In all patients Leak_conv _was higher than Leak_pause_, averaging 188 ± 159 ml (32 ± 25% of *V*_T,baseline_) and 61 ± 75 ml (10 ± 12% of *V*_T,baseline_), respectively (*P *< 0.05; Fig. 1). There was a significant linear relationship between Leak_conv _and Leak_pause _(*y *= - 12.3 + 0.39*x*, *r *= 0.84, *P *< 0.05; Fig. 1). The observed ΔLeak averaged 127 ± 105 ml. There was a significant linear relationship between Δ*P*_aw,peak _and the observed ΔLeak (*y *= 64.8 + 26.2*x*, *r *= 0.66, *P *< 0.05) and between the predicted and observed ΔLeak (*y *= 13.14 + 0.73*x*, *r *= 0.69, *P *< 0.05). There was no relationship between observed ΔLeak and respiratory system mechanics (*R*_int_, *R*_rs_, Δ*R *and *C*_rs_), the time constant of the respiratory system and *V*_T,baseline_.

### Model study

#### Protocol A

**Figure 2:**
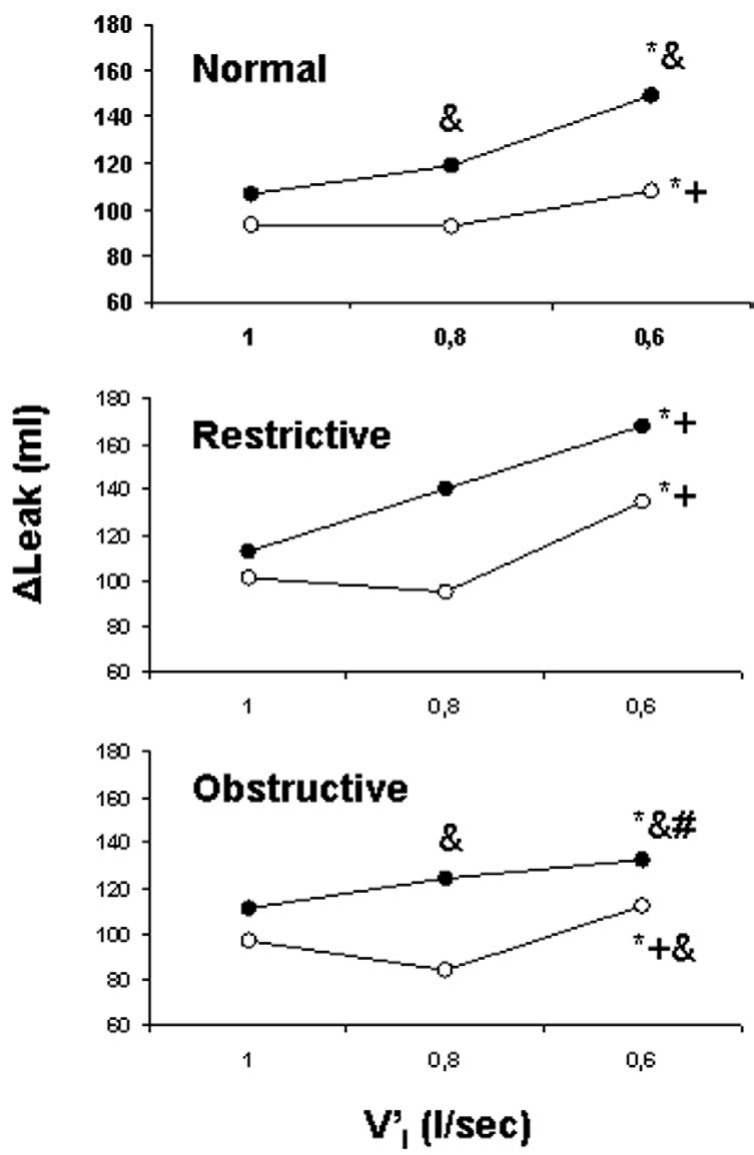
Lung model study, protocol I. ΔLeak (difference between Leak_conv _and Leak_pause_) is shown at given inspiratory flow (*V*'_I_) as a function of cross-sectional area around the endotracheal tube in a simulated model of respiratory system disease. Filled circles, large cross-sectional area; open circles, small cross-sectional area. *, Significantly different from the corresponding value at *V*'_I _= 1 litre/s. ^+^, Significantly different from the corresponding value at *V*'_I _= 0.8 litre/s. ^&^, Significantly different from the corresponding value for simulated restrictive respiratory system disease. ^#^, Significantly different from the corresponding value for simulated normal respiratory system.

For a given condition, Leak_conv _was significantly higher than Leak_pause _(Table 2). For a given cross-sectional area, and independently of model mechanics, Leak_pause _was not affected by *V*'_I_, whereas Leak_conv _increased significantly with decreasing *V*'_I _(Table 2). Independently of the cross-sectional area around the endotracheal tube with simulated restrictive respiratory system disease and at a *V*'_I _of 0.6 litre/s, Leak_conv _was significantly higher than the corresponding values with simulated normal mechanics and obstructive respiratory system disease. ΔLeak increased significantly with decreasing *V*'_I _and increasing the size of the cross-sectional area around the endotracheal tube (Fig. 2). The effect of *V*'_I _on ΔLeak was significantly higher with simulated restrictive respiratory system disease and large cross-sectional area around the endotracheal tube (Fig. 2).

#### Protocol B

**Figure 3:**
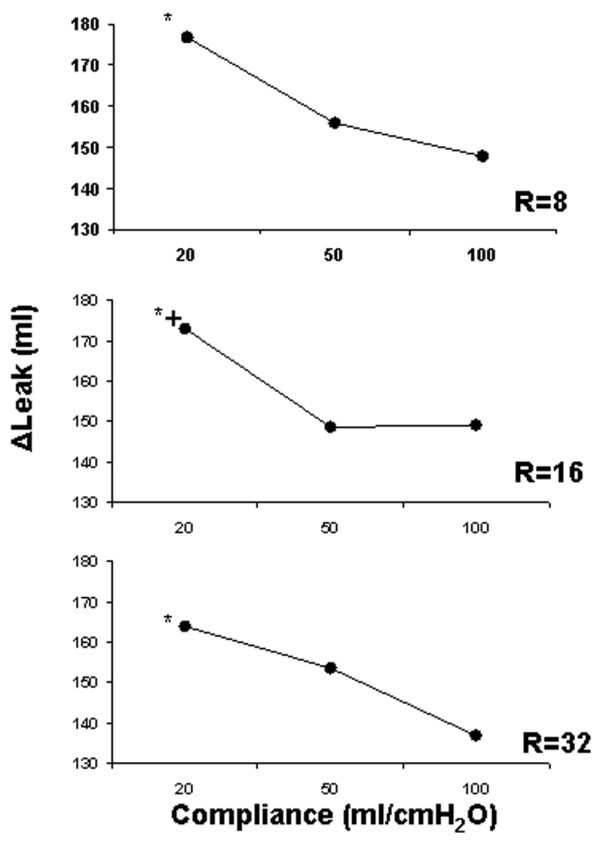
Lung model study, protocol II. ΔLeak (difference between Leak_conv _and Leak_pause_) is shown at constant inspiratory flow as a function of respiratory system mechanics in a simulated model of constant cross-sectional area around the endotracheal tube. *R*, model airway resistance (cmH_2_O/litre per second); *C*, model compliance (ml/cmH_2_O). *, Significantly different from the corresponding value at *C *= 100 ml/cmH_2_O. ^+^, Significantly different from the corresponding value at *C *= 50 ml/cmH_2_O.

Similarly to protocol A, and independently of model mechanics, Leak_conv _was significantly higher than Leak_pause _(Table 3). For a given *R*, Leak_conv _increased significantly with decreasing *C*, whereas Leak_pause _remained constant. For a given *C*, Leak_pause _and Leak_conv _tended to increase slightly with the highest resistance, the difference being significant only for Leak_pause_. ΔLeak was not affected by model resistance, whereas it increased significantly with decreasing compliance (Fig. 3).

## Discussion

The main findings of this study were as follows. First, because in mechanically ventilated patients the expiratory leak volume is about 30% of the sum of inspiratory and expiratory leaks (total leak), the inspiratory leak significantly affected the results of the cuff-leak test. Second, the cross-sectional area around the endotracheal tube is not the only determinant of cuff-leak test. Third, respiratory system compliance and inspiratory flow affect the test significantly, mainly through an effect on the inspiratory component. Fourth, the expiratory component is slightly influenced by respiratory system resistance.

To avoid the confounding factors of respiratory muscle activity and dynamic hyperinflation on the calculation of cuff-leak volume, the patients were paralyzed and ventilated with settings that permitted the respiratory system to reach passive functional residual capacity at the end of expiration. Similarly, in the lung model the ventilator settings were such that dynamic hyperinflation was not observed. Therefore, for a given experimental condition the inspired tidal volume entirely determined the total expired volume. Finally, contrary to other studies [5], cuff-leak volume was measured by comparing the expired tidal volume with and without a deflated cuff. In this case the difference between inspired and expired tidal volume due to gas exchange and the different temperature and humidity of inspired and expired gas were not an issue.

By deflating the cuff at the end of the inspiratory pause we guaranteed that the ventilator delivered all of the predetermined gas volume into the lung, as indicated by the similar peak *P*_aw _between the breaths used to calculate the cuff-leak volume. Because inactivity of respiratory muscles and absence of dynamic hyperinflation were ensured, any difference in expired volume with and without a deflated cuff should be entirely due to gas leak around the endotracheal tube during expiration (pause cuff leak). In contrast, when the cuff-leak volume was measured with the conventional method, a fraction of gas volume delivered by the ventilator might leak around the endotracheal tube during inspiration. In that case the measured cuff-leak volume is the total leak consisting of an inspiratory and expiratory component. The design of this study did not permit us to measure with accuracy the inspiratory leak. This is because pause cuff leak is not similar to expiratory leak obtained with the conventional method because end-inspiratory lung volume and thus elastic recoil pressure at the beginning of expiration differ substantially between the two methods of cuff leak determination. The pause cuff leak should be higher than the expiratory component of the total leak, because end inspiratory lung volume and elastic recoil pressure were considerably higher when pause cuff leak was obtained.

Both in clinical and model study the cuff-leak volume determined with the conventional method (Leak_conv_) was always higher than that obtained by cuff deflation at end-inspiratory pause, which eliminated the inspiratory component of total leak (Leak_pause_). It follows that the inspiratory component is an important determinant of the cuff-leak test. It is of interest to note that in patients Leak_conv _was about threefold Leak_pause _whatever the amount of the total leak.

In Protocol A of the lung model study, for a given cross-sectional area, the system mechanics and inspiratory flow considerably affected Leak_conv_; Leak_conv _increased significantly with decreasing compliance and inspiratory flow. In contrast, neither system compliance nor inspiratory flow influenced Leak_pause_, which remained relatively constant. As a result ΔLeak increased significantly with decreasing compliance and inspiratory flow. The constancy of Leak_pause _suggested that the expiratory component of the total leak was also unaffected by changes in system compliance and inspiratory flow. It follows that respiratory system compliance and inspiratory flow have an important impact on cuff-leak test, mainly through an effect on the inspiratory component. The increased inspiratory leak with decreasing system compliance is predictable because the stiffness of the respiratory system causes a greater fraction of inspiratory flow to deviate to atmosphere though the free space between the endotracheal tube and the trachea. Similarly, the increased inspiratory leak with low inspiratory flow was also expected. The free space between the endotracheal tube and trachea represents a low-resistance pathway and, because for a given tidal volume low inspiratory flow is associated with longer inspiratory time, the inspiratory leak should increase, a situation resembling that of bronchopleural fistula in which high inspiratory flows are recommended so as to reduce the amount of air leaking through the fistula [14]. Thus the cuff-leak volume calculated by the conventional method does not solely reflect the cross-sectional area of the trachea and/or the upper airways but is influenced by other factors such as respiratory system mechanics and inspiratory flow.

In protocol B of the lung model study, a slight increase in cuff-leak volume at the highest resistance value was observed with both methods. As a result, ΔLeak was not influenced by model resistance, indicating that system resistance affected mainly the expiratory component of the total leak. Although the factors underlying the above increase are not clear, the flow velocity profile during expiration could account for these findings. Nevertheless the difference was relatively small (less than 25 ml or less than 4% of *V*_T_), making the clinical significance of this finding questionable. Furthermore the increase in expiratory leak was observed at very high values of resistance that preclude the weaning process, making the performance of the cuff-leak test clinically irrelevant.

We should note that in patients the cuff leak was determined at the relatively high constant inspiratory flow of 1 litre/s. Although the effect of flow was not studied in our patients, the model study indicates that overestimation should be higher at low flow. Nevertheless, high inspiratory flow is recommended in patients with obstructive lung disease ventilated on volume control so as to reduce dynamic hyperinflation [15].

In contrast with the model study, in the clinical study there was no relationship between observed ΔLeak and respiratory system mechanics (*R*_int_, *R*_rs_, Δ*R *and *C*_rs_), the time constant of the respiratory system and *V*_T,baseline_. Differences in cross-sectional area of the trachea and upper airways between patients might obscure any relationship between these variables and ΔLeak.

Studies suggest that leak volume, as obtained by the conventional method, may predict the occurrence of post-extubation stridor and might thus identify the subset of patients at risk of re-intubation due to upper airway obstruction [4,5,7]. However, the cut-off point of leak volume differed substantially between studies. In addition, the positive predictive value was quite low, indicating that the results of the cuff-leak test should not be used to postpone the extubation but might be particularly useful to exclude significant laryngeal edema [4,5,7,16]. In contrast, other authors concluded that the cuff-leak test is inaccurate [8]. Indeed, a cuff-leak volume (measured conventionally) of more than 300 ml has been observed in three patients who developed post-extubation stridor after cardiac surgery [8]. Although these different results between studies might be due to the populations studied, our study indicates that the respiratory system mechanics and inspiratory flow, factors influencing the inspiratory leak that were not taken into account, might to some extent contribute to the poor performance of the cuff-leak test.

A measured conventional cuff-leak volume of less than 15.5% [4], 12% [7] or 10% of predetermined *V*_T _[6] has been used to identify patients at risk for post-extubation stridor. In our study with the conventional method, 5 of 15 patients had a cuff-leak volume less than 15.5% of predetermined *V*_T_, whereas with the pause method 11 patients demonstrated true cuff-leak volume less than this threshold (10 patients had a cuff-leak volume less than 12%). The purpose and design of our study were such that they did not permit us to examine whether by eliminating the inspiratory leak it would be possible to improve the predictive value of the cuff-leak test. The number of patients was small and the cuff-leak volume was not determined on the day of extubation, but the patients were examined under highly controlled conditions. The aim of the study was not to propose a new method of cuff leak determination but to examine factors affecting the total cuff-leak volume obtained by the conventional method. Our results clearly showed that the cuff-leak test (particularly its inspiratory component) is influenced by factors other than the cross-sectional area of the trachea and/or the upper airways and thus the above-mentioned cut-off points of cuff-leak volume should be re-evaluated.

## Conclusion

Our study has shown that the cross-sectional area around the endotracheal tube is not the only determinant of the cuff-leak test. Respiratory system mechanics and inspiratory flow are other important determinants of the cuff-leak test, mainly through an effect on the inspiratory component of the total leak, complicating its interpretation.

## Key messages

• 	Cross-sectional area around the endotracheal tube is not the only determinant of the cuff leak test.

• 	Respiratory system mechanics and inspiratory flow are the other important determinants of the cuff leak test, complicating its interpretation.

## Abbreviations

*C *= model airway compliance; *C*_rs _= end-inspiratory static compliance of the respiratory system (ml/cmH_2_O); ΔLeak = difference between Leak_conv _and Leak_pause_; Δ*P*_aw,peak _= difference between peak inspiratory *P*_aw _between methods; Δ*R *= difference between *R*_rs _and *R*_int_; Leak_conv _= cuff-leak volume obtained by the conventional method; Leak_pause _= cuff-leak volume obtained when the cuff was deflated at the end of the end-inspiratory pause; *P*_aw _= airway pressure; PEEP = positive end-expiratory pressure; *R *= model airway resistance; *R*_int _= minimum resistance of the respiratory system; *R*_rs _= maximum resistance of the respiratory system; *V*' = flow at the airway opening; *V*'_I _= inspiratory flow; *V*_T _= expired tidal volume; *V*_T,baseline _= expiratory *V*_T _measured by averaging five consecutive breaths; *V*_T,defl _= expiratory *V*_T _measured when cuff was deflated; *V*_T,pause _= expiratory tidal volume measured at the end of the end-inspiratory pause.

## Competing interests

The author(s) declare that they have no competing interests.

## Authors' contributions

GP designed the study and performed the statistics. CA collected the data from patients and from the model. EM and EK participated in data collection. DG designed the study, evaluated the data and drafted the manuscript. All authors read and approved the final manuscript.

## Figures and Tables

**Table 1:** Baseline ventilator settings and patients' respiratory system mechanics

No.	*V*_T_	Fr	*C*_rs_	*R*_int_	*R*_rs_
1	0.68	12.8	47.5	12.6	17.1
2	0.64	13.0	27.2	8.4	12.4
3	0.61	8.1	63.2	13.1	20.4
4	0.70	7.1	57.8	14.1	17.5
5	0.46	7.1	63.8	14.9	17.2
6	0.68	14.9	30.5	11.0	15.5
7	0.52	14.5	30.9	10.8	15.0
8	0.58	11.6	28.0	13.8	18.5
9	0.62	8.5	51.2	13.4	15.1
10	0.60	13.5	32.2	13.3	17.3
11	0.66	9.8	56.1	8.5	12.8
12	0.58	10.4	17.7	9.6	22.8
13	0.51	13.0	37.6	9.0	14.0
14	0.56	16.0	43.9	6.7	13.3
15	0.49	11.8	36.4	10.7	13.6
Mean	0.59	11.5	41.6	11.3	16.2
SD	0.07	2.9	14.4	2.5	2.9

*C*_rs_, end-inspiratory static compliance of the respiratory system (ml/cmH_2_O); Fr, ventilator frequency (breaths/min); *R*_int _and *R*_rs_, minimum and maximum inspiratory resistance (cmH_2_O/l per second), respectively; *V*_T_, tidal volume (litres).

**Table 2:** Model study: protocol A

Parameter	Normal pattern			Restrictive pattern			Obstructive pattern		
	*V*' = 1	*V*' = 0.8	*V*' = 0.6	*V*' = 1	*V*' = 0.8	*V*' = 0.6	*V*' = 1	*V*' = 0.8	*V*' = 0.6
Large area									
Leak_pause _(ml)	191 ± 7	196 ± 6	190 ± 4	190 ± 13	190 ± 15	190 ± 6	196 ± 5	185 ± 6	187 ± 6
Leak_conv _(ml)	298 ± 6	315 ± 3^a^	339 ± 4^ab^	303 ± 6	330 ± 2^a^	358 ± 2^ab^	308 ± 7	309 ± 5	320 ± 10^ab^
Small area									
Leak_pause _(ml)	146 ± 2	135 ± 5	135 ± 4	147 ± 8	148 ± 12	137 ± 4	146 ± 9	139 ± 6	141 ± 11
Leak_conv _(ml)	239 ± 7	228 ± 3	244 ± 4^ab^	249 ± 10	243 ± 4	269 ± 7^ab^	243 ± 14	234 ± 4	254 ± 6^ab^

Results are means ± SD. *V*', constant inspiratory flow (litre/s); Leak_conv_, cuff-leak volume measured when the cuff remained deflated during both inspiration and expiration; Leak_pause_, cuff-leak volume measured when the cuff was deflated at the end of 3 s of inspiratory pause.

^a^Significantly different from the corresponding value at *V*'_I _= 1 litre/s.

^b^Significantly different from the corresponding value at *V*'_I _= 0.8 litre/s.

**Table 3:** Model study: protocol B

Parameter	*R *= 8			*R *= 16			*R *= 32		
	*C *= 20	*C *= 50	*C *= 100	*C *= 20	*C *= 50	*C *= 100	*C *= 20	*C *= 50	*C *= 100
Leak_pause _(ml)	96 ± 9	99 ± 6	96 ± 9	105 ± 10	103 ± 11	110 ± 8	123 ± 12^c^	115 ± 9	118 ± 12^c^
Leak_conv _(ml)	275 ± 11^a^	257 ± 9	245 ± 8	278 ± 6^ab^	261 ± 10	253 ± 9	287 ± 13^ab^	268 ± 7	255 ± 6

Results are means ± SD. *C*, model compliance (ml/cmH_2_O); Leak_conv_, cuff-leak volume measured when the cuff remained deflated during both inspiration and expiration; Leak_pause_, cuff-leak volume measured when the cuff was deflated at the end of 3 s of inspiratory pause; *R*, model resistance (cmH_2_O/litre per second).

^a^Significantly different from the corresponding value at *C *= 100 ml/cmH_2_O.

^b^Significantly different from the corresponding value at *C *= 50 ml/cmH_2_O.

^c^Significantly different from the corresponding value at *R *= 8 cmH_2_O/litre per second.
